# SpTransformer proteins from the purple sea urchin opsonize bacteria, augment phagocytosis, and retard bacterial growth

**DOI:** 10.1371/journal.pone.0196890

**Published:** 2018-05-08

**Authors:** Hung-Yen Chou, Cheng Man Lun, L. Courtney Smith

**Affiliations:** Department of Biological Sciences, George Washington University, Washington, DC, United States of America; Institute of Oceanology, Chinese Academy of Sciences, CHINA

## Abstract

The purple sea urchin, *Strongylocentrotus purpuratus*, has a complex and robust immune system that is mediated by a number of multi-gene families including the *SpTransformer* (*SpTrf*) gene family (formerly *Sp185/333*). In response to immune challenge from bacteria and various pathogen-associated molecular patterns, the *SpTrf* genes are up-regulated in sea urchin phagocytes and express a diverse array of SpTrf proteins. We show here that SpTrf proteins from coelomocytes and isolated by nickel affinity (cNi-SpTrf) bind to Gram-positive and Gram-negative bacteria and to Baker’s yeast, *Saccharomyces cerevisiae*, with saturable kinetics and specificity. cNi-SpTrf opsonization of the marine bacteria, *Vibrio diazotrophicus*, augments phagocytosis, however, opsonization by the recombinant protein, rSpTrf-E1, does not. Binding by cNi-SpTrf proteins retards growth rates significantly for several species of bacteria. SpTrf proteins, previously thought to be strictly membrane-associated, are secreted from phagocytes in short term cultures and bind *V*. *diazotrophicus* that are located both outside of and within phagocytes. Our results demonstrate anti-microbial activities of native SpTrf proteins and suggest variable functions among different SpTrf isoforms. Multiple isoforms may act synergistically to detect a wide array of pathogens and provide flexible and efficient host immunity.

## Introduction

Sea urchins are basal members of the deuterostome lineage of animals and belong to the echinoderm phylum, which is sister to the chordates that includes vertebrates. This close relationship makes purple sea urchins optimal organisms for investigations of fundamental functions and evolutionary diversity of innate immunity in deuterostomes. There is a wide range of solutions among organisms for detecting and combating pathogens and opportunists that employ various sequence diversification mechanisms for immune proteins [1]. Examples include several versions of the clustered regularly interspaced palindromic repeats (CRISPR) and CRISPR-associated (Cas) protein immune system in bacteria and archaea that provide protection against re-infection by bacteriophages [2], a large expansion of *C1q* genes in the Pacific oyster genome [3] with binding function against pathogen-associated molecular patterns (PAMPs) [4], somatic diversification of the multigene family encoding fibrinogen related proteins (FREPs) in molluscs that opsonize parasites [5–7], a family of genes encoding variable domain-containing chitin binding proteins (VCBPs) in protochordates [8] that respond to gut microbes [9], extensive alternative splicing of the single copy gene encoding Down syndrome cell adhesion molecules (DSCAM) in arthropods [10, 11] that function in anti-microbial and anti-viral responses [12, 13], the assembled variable lymphocyte receptors (VLRs) in cyclostomes that bind a wide range of foreign targets [14], and somatic rearrangement of the immunoglobulin (*Ig*) gene family in higher vertebrates [15, 16] that recognize intact pathogens and processed antigen fragments. Furthermore, there are expanded families of Toll-like receptor (*TLR*) genes and NOD-like receptor (*NLR*) genes in the genomes of sea urchins and amphioxus [17, 18], expansion of disease resistance (*R*) gene families in higher plants [19, 20], hundreds of alleles for the single copy fusion/histocompatibility (*Fu/HC*) gene in populations of compound tunicates [21] and the family of major histocompatibility complex (*MHC*) genes in humans [22] that function at the core of self/non-self recognition. Despite the wide range of mechanisms to diversify immune genes in the arms race with pathogens, from which successful pathogens are selected based on abilities to avoid or overcome the host immune system, invertebrates and plants generally rely solely on innate responses for host protection. The innate immune system of the purple sea urchin, *Strongylocentrotus purpuratus*, was first characterized as complex through genome and transcriptome annotation [23–26] and is likely an important physiological attribute of this large, long-lived invertebrate [27] that is in continual contact with multitudes of marine microbes both on the external epithelia and the mucosal surfaces of the gut for both the adults and larvae [28]. In addition to the large sizes of the *SpTLR* and *SpNOD* gene families in the purple sea urchin [17, 18], immune genes in this invertebrate also include those that encode small lectins [25], a complement system [29] that may be activated by homologues of mannose binding lectin and/or ficolins [25, 30], cysteine-rich scavenger receptors [31], peptidoglycan (PGN) recognition proteins, as well as Gram-negative binding proteins [25] and anti-microbial peptides [32, 33]. Pathogen detection in the purple sea urchin also induces expression of the *SpTransformer* (*SpTrf*) gene family (previously called the *Sp185/333* family) [34–36]. *HeTrf* genes are expressed similarly in another sea urchin species, *Heliocidaris erythrograma* [37] and orthologous genes are present in the genome sequences of other echinoid families except for the cidaroid family or pencil sea urchins. The sequenced genome of the purple sea urchin has three clusters of tightly linked *SpTrf* genes for a total of 15 genes [38, 39].

**Fig 1:**
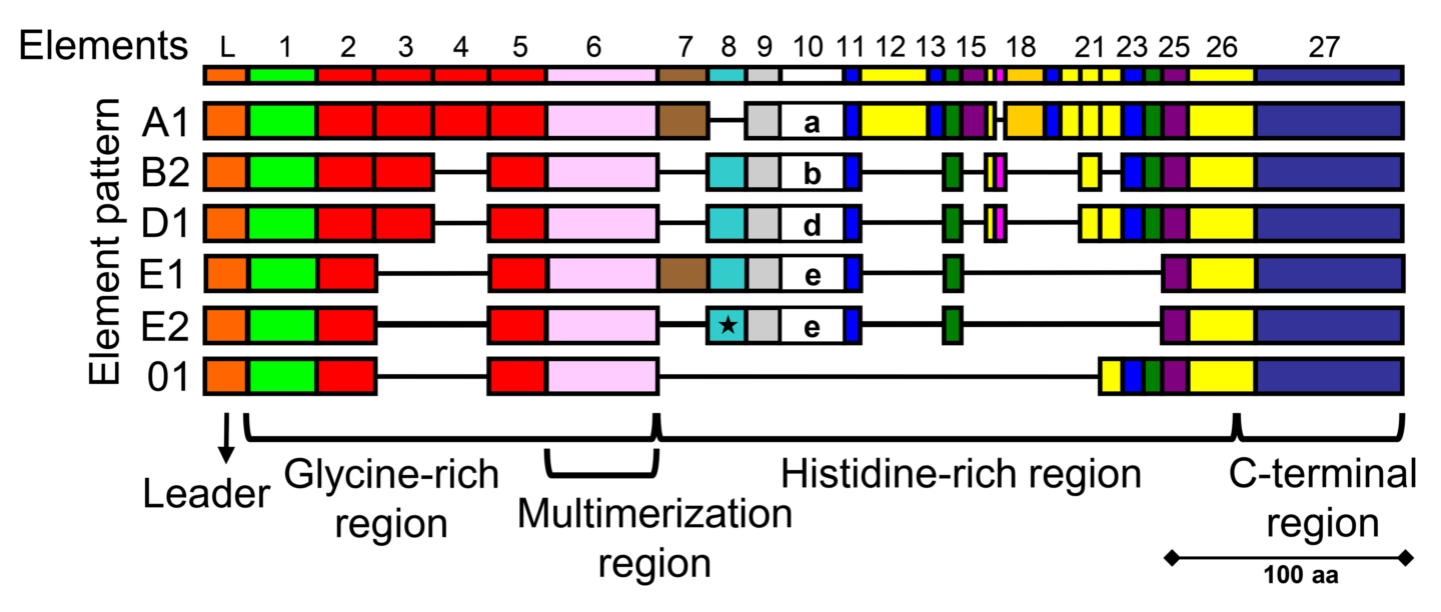
The SpTrf proteins are highly variable. Examples of different SpTrf protein isoforms are shown as a cartoon alignment. All proteins include a hydrophobic leader and the mature protein, which is a mosaic of sequence blocks or elements (indicated as colored rectangles) that make up mosaics of element patterns. All possible elements are shown at the top, although the SpTrf proteins never include all possible elements. The patterns correlate with the sequence of element 10 that is highly diverse and is used to define the pattern type [36]. Mature SpTrf proteins consist of a glycine-rich region with a multimerization region, a histidine-rich region, and a C-terminal region. rSpTrf-E1 has an E1 element pattern, which has been rarely identified. Some proteins are truncated as an outcome of RNA editing that generates frameshifts and inserts stop codons [54], the most common of which is an edit that creates a stop codon in element 8 (indicated by the star) for the E2 pattern. This figure is modified from Buckley & Smith [48].

A subset of the sea urchin immune cells, the phagocytes, express the *SpTrf* genes. The immune cells in the adult, called coelomocytes, are present in the coelomic cavity. In larvae, derivatives of the non-skeletogenic mesenchyme (NSM) cells located in the blastocoel. In both adults and larvae, the immune cells are complex sets of different cell types that can be activated with lipopolysaccharide (LPS) and other PAMPs in addition a variety of bacteria [28, 35, 40–44]. Immune activation of adult coelomocytes and larval NSM derivatives are particularly responsive to the marine microbe, *Vibrio diazotrophicus*, that was originally isolated from the gut of the green sea urchin, *S*. *droebachiensis* [45]. *SpTrf* genes are expressed by three types of phagocytes of adult sea urchins and the blastocoelar cells in larvae [43, 46, 47]. The genes have two exons of which the first encodes the leader and the second encodes highly variable mature proteins [36, 48] that are composed of blocks of sequences called *elements* present in mosaics known as *element patterns* (Fig 1) [35, 36, 48]. The element patterns impart significant sequence diversity among the proteins, however, they have a standard overall structure of a glycine-rich region with multimerization function, a histidine-rich region, and a C-terminal region (Fig 1) [36, 49]. Other anti-microbial peptides (AMPs) are characterized by elevated abundances of a particular amino acid, such as clavanin A that is enriched in histidine, glycine, and phenylalanine or proline [50] and other extended AMPs that are rich in arginine and other residues (reviewed in [51]). The SpTrf protein diversity in individual sea urchins responding to immune challenge has been evaluated by two dimensional Western blot showing an estimate of up to 260 distinct SpTrf-positive spots indicating a large array of protein isoforms plus significant multimerization [52]. Because individual sea urchins express variant arrays of SpTrf proteins that change in response to the same and/or different immune challenges, the diversity of the SpTrf protein arrays within the population is expected to be very large [53]. Although the functions of the SpTrf proteins are not fully understood, the diversity of the deduced amino acid sequences and their expression kinetics suggest that they act as immune effectors, and predictions suggest that different variants may have different functions [52].

A recombinant SpTrf protein, rSpTrf-E1 (originally called rSp0032), with an E1 element pattern (Fig 1) binds tightly and specifically to *Vibrio diazotrophicus* and to *Saccharomyces cerevisiae*, but does not bind to two *Bacillus* species [49]. rSpTrf-E1 binds tightly to LPS, β-1,3-glucan, and flagellin, which is consistent with binding to yeast and Gram-negative bacteria. However, it does not bind to PGN, which is consistent with failure to bind to the Gram-positive *Bacillus* species. The range of cellular targets and PAMPs to which rSpTrf-E1 binds, and those to which it does not bind, is unexpected for an anti-pathogen protein and suggests unusual and flexible binding mechanisms. rSpTrf-E1 is an intrinsically disordered protein (IDP) based on bioinformatic predictions and results from circular dichroism, however the secondary structure transforms from disordered to α helical in the presence of binding targets such as LPS, the phospholipid, phosphatidic acid (PA), and sodium dodecyl sulfate or 2,2,2-trifluoroethanol that are typically used in circular dichroism for evaluating the secondary structure of proteins [55, 56]. Once rSpTrf-E1 binds to a target, K_d_ values are consistent with an off-rate that is below measurable levels, and tight binding may be the outcome of both initial target association followed by structural transformation. The notion of IDPs acting as anti-pathogen proteins that enables binding by individual isoforms to several PAMPs and foreign cells suggests a highly diverse and flexible response system for immune protection in echinoids.

Whole coelomic fluid (wCF) is a complex tissue consisting of a fluid matrix and four categories of coelomocytes that mediate the innate immune response to injury and infections through opsonization, agglutination, encapsulation, and phagocytosis [44, 57]. Activation of the coelomocytes through immune challenge increases the number of phagocytes containing SpTrf proteins and increases the amount of SpTrf proteins in the wCF [35, 46, 53, 58]. Furthermore, *SpTrf* mRNAs amplified from single phagocytes have identical sequences inferring expression of a single gene per cell [47], although predicted mRNA editing and post-translational modifications would suggest the production of a small set of similar proteins [52, 53, 59]. The proteins are localized to the membranes of perinuclear vesicles of all phagocyte types and are associated with the outer surface of the plasma membrane on small phagocytes [46, 47, 60]. This localization may be based on interactions between the SpTrf proteins and theoretical cell surface receptors or the histidine-rich region association with negatively charged molecules as predicted from rSpTrf-E1 binding activities [49, 56]

Although binding characteristics have been reported for rSpTrf-E1 [49, 56], only preliminary results are available for the activities of native SpTrf proteins isolated from coelomic fluid. Full-length SpTrf proteins with sufficient numbers of histidines can be isolated by nickel affinity from wCF, and they show variations among individual sea urchins, and bind variably to foreign cells [49, 53]. Whether SpTrf protein binding to microbes has functional opsonization activities to augment phagocytosis by phagocytic coelomocytes is not known. Here we expand on preliminary results and report on the binding and opsonization functions of SpTrf proteins isolated either from pelleted coelomocytes or wCF from individual sea urchins. The SpTrf proteins isolated by nickel affinity from pelleted coelomocytes (cNi-SpTrf; see Table 1 for abbreviation definitions) or from wCF (wNi-SpTrf proteins) have a wide range of binding characteristics towards Gram-positive and Gram-negative bacteria. The cNi-SpTrf proteins isolated from different animals and rSpTrf-E1 show variable binding towards different bacteria and variable growth retardation for some bacterial species. When *Vibrio diazotrophicus* is opsonized with cNi-SpTrf and then incubated with sea urchin phagocytes, phagocytosis is augmented. Furthermore, phagocytes secrete SpTrf proteins into the medium in short term cultures that bind to heat-killed *V*. *diazotrophicus*, which are observed both in perinuclear phagosomes and outside of the phagocytes. Although rSpTrf-E1 binds tightly to *V*. *diazotrophicus* and LPS [49], it does not induce phagocytosis of opsonized *V*. *diazotrophicus* by coelomocytes. These results demonstrate broad activities of mixtures of SpTrf proteins compared to individual recombinant proteins, and suggest putative overlapping ranges of binding activities among individual SpTrf protein isoforms and that some, but not all induce phagocytosis. This innate effector response in sea urchins appears to be a dynamic system in which the protein isoforms likely function synergistically resulting in a highly responsive and flexible response to a wide range of pathogens.

**Table 1:** SpTrf protein abbreviations.

Abbreviation	Definition
wNi-SpTrf	SpTransformer proteins isolated by nickel affinity from lysates of whole coelomic fluid (wCF)
cNi-SpTrf	SpTransformer proteins isolated by nickel affinity from lysates of pelleted coelomocytes
*Vibrio*-FITC	*Vibrio diazotrophicus* stained with fluorescein isothiocyanate
cfCF-*Vibrio*-FITC	*Vibrio*-FITC opsonized with cell free coelomic fluid (cfCF)
aCF-*Vibrio*-FITC	*Vibrio*-FITC opsonized with artificial coelomic fluid (aCF); buffer control
cNi-SpTrf-*Vibrio*	*Vibrio* opsonized with cNi-SpTransformer proteins
FITC-cNi-SpTrf-*Vibrio*	Biotinylated cNi-SpTrf-*Vibrio* incubated with NeutrAvidinFITC
rSpTrf-E1-*Vibrio*	*Vibrio* opsonized with rSpTrf-E1
FITC-rSpTrf-E1-*Vibrio*	Biotinylated rSpTrf-E1-*Vibrio* incubated with NeutrAvidinFITC

## Materials and methods

### Sea urchins

Adult purple sea urchins, *Strongylocentrotus purpuratus*, were purchased from Marinus Scientific or the Southern California Sea Urchin Company after collection from the coast of southern California. Animals were maintained as previously described [61].

### Preparation of bacteria and *Saccharomyces cerevisiae*

*Vibrio diazotrophicus* was purchased from the American Type Culture Collection (item #33466). *Bacillus cereus* and *B*. *subtilis* were a gift from David Morris (George Washington University). *Escherichia coli* were similar to DH10B (One Shot^®^ bacteria; Invitrogen), and *Saccharomyces cerevisiae* was type II (Sigma-Aldrich). Cultures were grown as previously described [49, 53, 62, 63]. Bacteria and yeast were heat-killed, washed, and resuspended in artificial coelomic fluid (aCF; 10 mM CaCl_2_, 14 mM KCl, 50 mM MgCl_2_, 398 mM NaCl, 1.7 mM Na_2_HCO_3_, 25 mM Na_2_SO_4_). *Bacillus cereus*, *B*. *subtilis*, and *S*. *cerevisiae* were counted using TC20^™^ Automated Cell Counter (Bio-Rad Laboratories). To facilitate counting of heat-killed *E*. *coli* and *V*. *diazotrophicus*, an aliquot of each were stained with fluorescein isothiocyanate (FITC; 0.01mg/ml aCF; Fisher Scientific) at 37°C for 30 min, washed at least seven times with aCF, and counted with a hemocytometer using the green fluorescence channel on an Axioplan fluorescence microscope (Zeiss). The concentrations of the heat-killed cells, including FITC-stained *Vibrio diazotrophicus* (*Vibrio*-FITC), were adjusted to 1 X 10^8^ cells per ml in aCF and aliquots of 250 μl were stored at 4°C.

### Immune challenge and preparation of cell-free coelomic fluid

Sea urchins were challenged with an initial injection of 1 X 10^4^ heat-killed *Vibrio diazotrophicus* per ml of coelomic fluid (CF, volume per animal was estimated according to [40] and injected a second or a third time at 24 and 48 hr with 1 X 10^6^ heat-killed *V*. *diazotrophicus* per ml of CF as described [53]. Animals were sacrificed 24 hr after the last injection by removing Aristotle’s Lantern and collecting the wCF in 50 ml tubes on ice, which were gently inverted several times to allow the wCF to clot. The wCF was centrifuged (Eppendorf, model 5415C) at 10,000 x *g* at 4°C for 5 min and the supernatant, or cell free CF (cfCF), was stored at -70°C in 1 ml aliquots for use in opsonization assays (see below). The pellet was either used to isolate SpTrf proteins (see below) or was discarded.

### Isolation of rSpTrf-E1 and SpTrf proteins by nickel affinity

rSpTrf-E1 was expressed in *E*. *coli*, isolated, and purified by nickel affinity as described [49]. SpTrf proteins were isolated from wCF lysates by affinity to Ni-His60 resin (CloneTech Laboratories) according to the manufacturer and constituted the wNi-SpTrf proteins. Alternatively, SpTrf proteins were isolated from pelleted coelomocytes by nickel affinity employing optimized isolation methods according to Sherman et al. [53]. Briefly, wCF was collected without anticoagulant and allowed to clot, followed by pelleting both coelomocytes and clots. Pellets were resuspended in aCF plus protease inhibitors and detergents, lysed by sonication, centrifuged, and the supernatant was passed through a Ni-His60 column. Elution fractions containing the SpTrf proteins were identified by Western blot (see below) and constituted the cNi-SpTrf proteins.

### cNi-SpTrf and rSpTrf-E1 binding to target cells

Increasing concentrations of cNi-SpTrf proteins were mixed with a fixed number of *V*. *diazotrophicus* (6.2 X 10^9^ cells), *Bacillus cereus* (1.5 X 0^7^ cells), *B*. *subtilis* (1.5 X 10^7^ cells) or *Saccharomyces cerevisiae* (2.8 X 10^5^ cells) in 500 μl of aCF and rotated for 30 min at 4°C. Cells were pelleted (10,000 x *g* for 5 min) and resuspended in aCF twice followed by evaluation by Western blot (see below) for bound cNi-SpTrf proteins.

### Western blots

Bacteria with bound SpTrf proteins or samples of the nickel column eluates were heated to 95°C in SDS lysis buffer and separated by electrophoresis on a 12% sodium dodecyl sulfate—polyacrylamide gel (SDS-PAGE), transferred to PVDF membranes, and evaluated with anti-SpTrf antibodies according to [46, 49]. The sea urchin complement C3 homologue, SpC3, was detected using anti-SpC3-6H as described [53, 64, 65]. The antibody against SpC3 was raised to a 50 kDa recombinant fragment of the N terminal region of the SpC3 α chain [64]. Secondary antibodies were labeled with horse radish peroxidase, and blots were processed for ECL chemiluminescence (Thermo Fisher Scientific) followed by imaging with a ChemiDoc XRS+ (Bio-Rad Laboratories).

### Coelomocyte preparation

Coelomocytes were withdrawn as previously described [63, 66] with modifications. A 1 ml syringe with a 23-guage needle was preloaded with 300 μl of ice cold calcium-magnesium-free seawater containing 70 mM EDTA and 20 mM HEPES buffer (CMFSW-EH; 460 mM NaCl, 10.73 mM KCL, 7.04 mM Na_2_SO_4_, 2.38 mM NaHCO_3_, pH 7.4) and inserted through the peristomial membrane into the coelomic cavity from which 200 μl of wCF was withdrawn. The cells were immediately dispensed into 500 μl of ice cold CMFSW-EH and counted with a hemocytometer. Coelomocytes (either 1 X 10^4^, 2 X 10^4^, or 3 X 10^4^ in CMFSW-EH) were loaded into one of three cytology three-slot chimneys (Eppendorf) assembled with a poly-L-lysine coated slide (Polysciences or Newcomer Supply), and the volume in each chimney was adjusted to 200 μl with CMFSW-EH. The cytology chimney-slide assemblies were centrifuged at 1,000 x *g* for 5 min at 4°C (Eppendorf, model 5810R). After centrifugation, chimney-slide assemblies were held at 4°C for 5 min followed by incubation at 14°C on a cold plate attached to a circulating chiller (NESLAB RTE-211, Cole-Parmer). Damp paper towels were placed between the cold plate and the chimney-slide assemblies to maintain the slide temperature at 14°C. The supernatant in the chimney was gently aspirated and replaced with 200 μl of coelomocyte culture medium (CCM; 0.5 M NaCl, 5 mM MgCl_2_, 1 mM EGTA, 20 mM HEPES pH 7.4 [46, 67]). Cells were incubated in chimney-slide assemblies on the cold plate at 14°C for phagocytosis assays (see below).

### Opsonization and phagocytosis

wNi-SpTrf and cNi-SpTrf proteins were purified, concentrated and stored at 4°C for up to two weeks prior to use. Assays employed cNi-SpTrf and wNi-SpTrf proteins that were biotinylated with EZ-link Sulfo-NHS-LC-LC-Biotin (Thermo Fisher Scientific) at 14°C on a rotator and dialyzed three times for 2 hr each against 1 L of PBS at 14°C according to [49]. Opsonization assays were performed as described [63] with modifications. *Vibrio diazotrophicus* cells were opsonized with either biotinylated cNi-SpTrf (cNi-SpTrf-*Vibrio*) or with biotinylated rSpTrf-E1 (rSpTrf-E1-*Vibrio*; see Table 1 for abbreviations) by incubating 250 μl of *V*. *diazotrophicus* (1 X 10^8^ cells per ml) with 250 μl of either of cNi-SpTrf (2 μg/250 μl) or rSpTrf-E1 (1 μg/250 μl) for 40 min at 4°C on a rotator. The positive control was *Vibrio*-FITC (1 X 10^8^ cells per ml) in 250 μl of aCF incubated with 250 μ l of cfCF from a donor animal (cfCF-*Vibrio*-FITC) and rotated for 40 min at 4°C. The negative control was *Vibrio*-FITC (250 μl; 1 X 10^8^ cells per ml) incubated with 250 μl of aCF (aCF-*Vibrio*-FITC) for 40 min at 4°C. cfCF-*Vibrio*-FITC, aCF-*Vibrio*-FITC, cNi-SpTrf-*Vibrio*, and rSpTrf-E1-*Vibrio* were washed once with CCM and resuspended in 250 μl of CCM. NeutrAvidin-FITC (0.1%; Invitrogen) was linked to the biotinylated cNi-SpTrf-*Vibrio* and rSpTrf-E1-*Vibrio* according to the manufacturer. Opsonized *V*. *diazotrophicus* (FITC-cNi-SpTrf-*Vibrio*, FITC-rSpTrf-E1-*Vibrio*, cfCF-*Vibrio*-FITC, and aCF-*Vibrio*-FITC,) were added to coelomocytes on slides in the chimney-slide assemblies (see above) at a ratio of 100 *V*. *diazotrophicus* cells per coelomocyte and incubated for 70 min at 14°C. For some experiments, coelomocytes were incubated with non-opsonized, heat-killed *V*. *diazotrophicus* for 24 hr. Afterwards, the CCM in the chimneys was gently aspirated and each chimney was washed once with 200 μl of CCM. The chimney-slide assemblies were disassembled and the cells were processed for immunofluorescence imaging (see below). Phagocytosis was evaluated based on the percentage of coelomocytes (n > 500) that had phagocytosed *V*. *diazotrophicus*. The mean number of *V*. *diazotrophicus* cells phagocytosed per coelomocyte (n ≥ 500) was calculated for each sample. Three replicates of coelomocytes from three sea urchins were evaluated for phagocytosis of *V*. *diazotrophicus*. The statistical significance among samples and controls was determined using Student’s one-tailed *t* test or two-sample proportional t test.

### Coelomocyte processing for immunofluorescence

Coelomocytes and *V*. *diazotrophicus* on slides (see above) were processed for immunofluorescence imaging as described [46]. Briefly, cells were fixed, permeabilized, blocked, and incubated with primary antibodies to SpTrf proteins and actin followed by secondary antibodies labeled with fluorchromes (Pierce-Thermo Fisher Scientific), and nuclear DNA staining with 4’,6-diamidino-2-phenylindole (DAPI). Confocal imaging was performed with an LSM 710 confocal microscope (Zeiss) at the George Washington University Center for Microscopy and Image Analysis. Some images had false colors applied for better visualization.

### ELISA

ELISA assays were carried out according to Lun et al. [49] and Majeske et al. [58]. Briefly, 100 μl of LPS (2 μg/ml in standard PBS, #L4524 from *E*. *coli*, Sigma-Aldrich) was distributed into the wells of a 96-well ELISA plate (Corning Life Sciences) and incubated overnight followed by drying at 60°C. The amount of LPS bound to the wells was quantified with a *Limulus* amoebocyte lysate (LAL) chromogenic endotoxin kit (Pierce-Thermo Fisher Scientific) according to the protocol from the manufacturer. Wells were incubated with blocking solution (4% bovine serum albumin in standard PBS) followed by washing with PBS with 0.5% Tween-20 (PBST). rSpTrf-E1 of varying concentrations was pre-incubated with a mixture of three anti-SpTrf antisera (1:1000 dilution in blocking solution) for 2 hr at room temperature. The mixture was added to the wells coated with LPS, and incubated at room temp for 2 hr. Positive controls omitted the antisera in the preincubation step, and background controls omitted rSpTrf-E1 and/or LPS. Wells were washed with PBST and incubated with an equal mixture of three anti-SpTrf antisera (1:1000 dilution in blocking solution) followed by a secondary antibody conjugated with alkaline phosphatase (1:15000 dilution; Sigma-Aldrich). The color was developed by the substrate reaction with *p*-nitrophenyl phosphate (1 mg/ml in diethanolamine) and read at 405 nm on a Spectra MAX microplate spectrophotometer (Molecular Devices). Background results were subtracted from protein binding data. Results were analyzed with Student’s two-tailed paired *t* test for samples with equal variance with significance set at *p* ≤ 0.05.

### Turbidity assay for bacterial growth

*Vibrio diazotrophicus*, *E*. *coli*, *B*. *cereus* and *B*. *subtilis* were grown (see above) to OD_600_ = 1.0 and 10 μl of the each culture was mixed with increasing concentrations of either cNi-SpTrf proteins isolated from three different sea urchins or with rSpTrf-E1 and distributed in triplicate into wells of a 96-well flat bottom plate (Thermo Fisher Scientific). The final volume was adjusted to 200 μl with either standard Luria Bertani (LB) broth or Marine Broth (MB) depending on the bacterial species and according to [53]. Bacteria were incubated at room temperature and turbidity was recorded at 0, 2 and 4 hr by absorbance at OD_600_ using a Synergy HT Multi-Mode Microplate Reader (BioTek) as controlled by Gen5 Data Analysis Software (BioTek). Cultures without added SpTrf proteins served as positive controls. Wells with LB or MB without bacteria served as blanks, which were subtracted from bacterial growth data. Statistical significance was determined by two-way ANOVA with significance set at *p* ≤ 0.05.

## Results

### wNi-SpTrf proteins bind to bacteria and yeast

**Fig 2:**
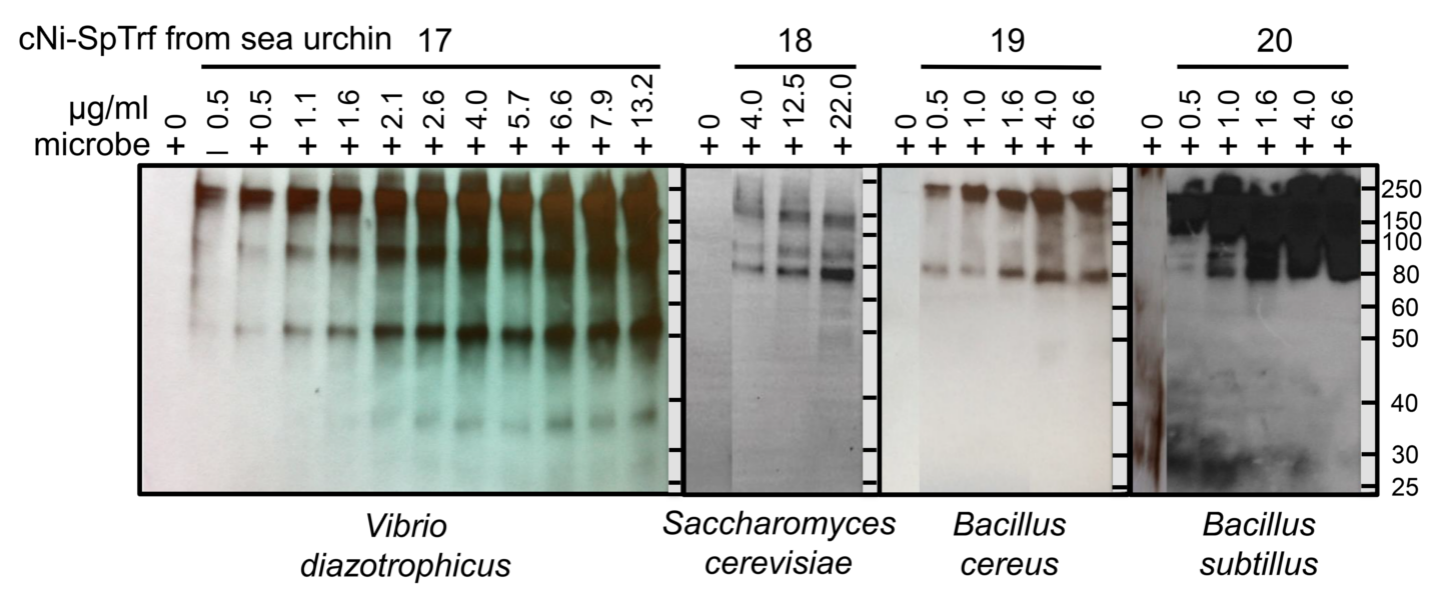
cNi-SpTrf proteins bind to bacteria and yeast. *Vibrio diazotrophicus* (6.2 X 10^9^ cells), *Saccharomyces cerevisiae* (2.8 X 10^5^ cells), *Bacillus cereus* (1.5 X 10^7^ cells), or *Bacillus subtilis* (1.5 X 10^7^ cells) were incubated with increasing amounts of cNi-SpTrf proteins isolated from four different sea urchins. Cells were washed, and analyzed by Western blot with anti-SpTrf antibodies. cNi-SpTrf proteins from each sea urchin bind to all target cells, but the patterns of the bands are different. Negative controls omitted the cNi-SpTrf proteins (0 μg) showing that neither the anti-SpTrf antibodies nor the secondary antibody bind directly to the bacteria or yeast. SpTrf proteins loaded directly onto the gel without pre-binding to *V*. *diazotrophicus* (**‒**, second lane for sea urchin 17) shows the pattern of bands. The first lane (0 μg/ml) of the blots for animals 18, 19, and 20, was moved closer to the lanes that show binding by the cNi-SpTrf proteins. This was done by digital methods to reduce the size of the figure and to make the comparisons among the lanes more clear. The blots were otherwise unaltered. Protein size standards in kDa are indicated to the right of each blot.

The SpTrf proteins expressed in sea urchins vary significantly among individuals and over time in response to immune challenge [53], and different isoforms within the arrays of SpTrf proteins have been speculated to impart a wide range of functions [49, 52]. Based on the numbers of histidines that are present in the histidine-rich region of the proteins (Fig 1), a subset of full-length SpTrf proteins can be isolated from wCF by nickel affinity [53]. Preliminary results indicate that wNi-SpTrf proteins (see Table 1 for abbreviation definitions) bind to a variety of foreign cells that varies depending on the individual sea urchin from which the proteins were isolated [49]. For a more detailed evaluation of the binding properties of the SpTrf proteins, cNi-SpTrf proteins were isolated from four sea urchins and evaluated for binding to *Vibrio diazotrophicus*, *Saccharomyces cerevisiae*, *Bacillus subtilis*, and *B*. *cereus*. Increasing amounts of cNi-SpTrf proteins correlated with increased binding to bacteria and yeast (Fig 2). At higher concentrations of cNi-SpTrf proteins, the intensities of the bands on Western blot lanes appeared similar suggesting that saturation binding had been reached. In an alternative approach to evaluate FITC-rSpTrf-E1 binding to *V*. *diazotrophicus* or *S*. *cerevisiae* by cell fluorescence using a plate reader, results were in agreement with saturation binding by Western blot (S1 Fig), and as reported previously using flow cytometry [49]. The patterns and intensity of SpTrf-positive bands on Western blots from four sea urchins differed for the different species of bacteria and for yeast to which they bound (Fig 2). cNi-SpTrf proteins that bound to *V*. *diazotrophicus* showed a much broader size range compared to those that bound to *S*. *cerevisiae* and to the *Bacillus* species. These results indicated that the cNi-SpTrf proteins and their binding activities differed among animals, in agreement with findings reported previously for wNi-SpTrf proteins isolated from the wCF collected from 18 sea urchins [53].

**Fig 3:**
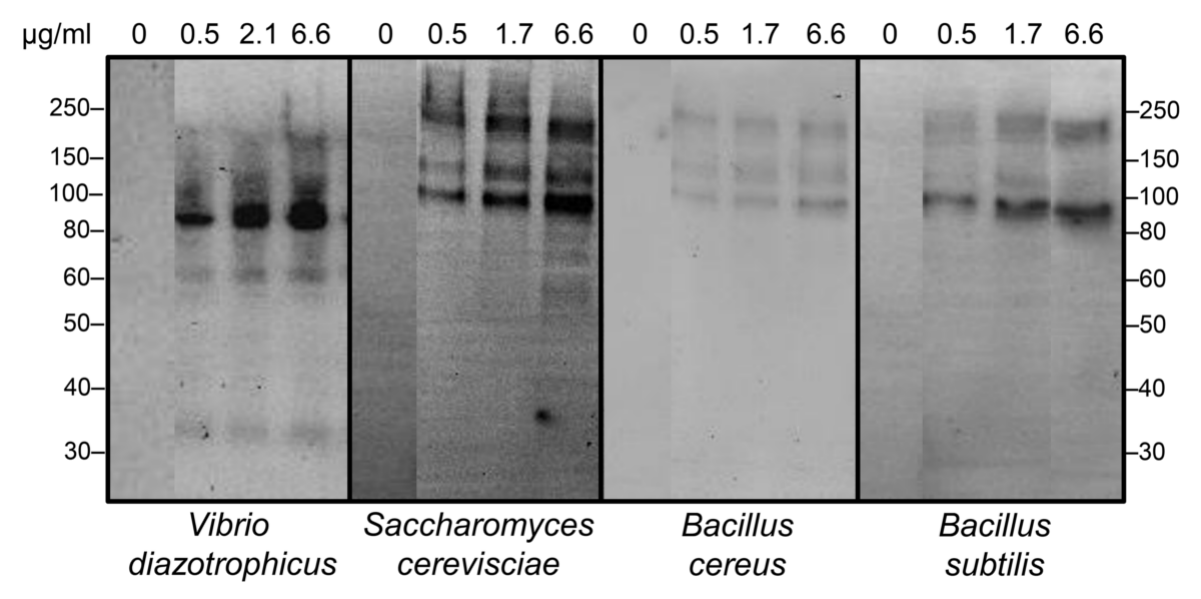
cNi-SpTrf proteins isolated from a single sea urchin bind variably to *Vibrio diazotrophicus*, *Saccharomyces cerevisiae*, *Bacillus cereus*, and *B*. *subtilis*. cNi-SpTrf proteins (μg/ml) were incubated with bacteria and yeast and washed cells were evaluated with multiple Western blot using the identical protocol including imaging timing. Negative controls omitted the cNi-SpTrf proteins (0 μg/ml). The first lane in each image was moved closer to the lanes that show binding by the cNi-SpTrf proteins. This was done by digital methods to reduce the size of the figure and to make the comparisons among the lanes more clear. The blots were otherwise unaltered. Protein size standards in kDa are indicated on the left and right of the blots.

The combination of variability for the cNi-SpTrf protein arrays among individual sea urchins [52, 53] and variability in binding activity towards target cells illustrated significant complexity in this system, which may be of benefit to sea urchin populations in the arms race against microbes. However, to simplify the experimental approach somewhat, cNi-SpTrf proteins were isolated from a single sea urchin and binding to the same four types of target cells was repeated. Results showed that the cNi-SpTrf proteins that bound to *V*. *diazotrophicus* consisted of a different range in molecular weight (major bands at ~60 and ~80 kDa) plus what was likely a monomer (~35 kDa), compared to the greater range of protein sizes bound to *S*. *cerevisiae* and the *Bacillus* species (~85 to ~200 kDa) (Fig 3). Overall, the arrays of cNi-SpTrf proteins produced by different sea urchins appeared to have different binding characteristics towards different foreign cells and that individual sea urchins may express different SpTrf isoforms for binding to different microbes.

### *Vibrio diazotrophicus* opsonized with cNi-SpTrf proteins enhances phagocytosis

**Fig 4:**
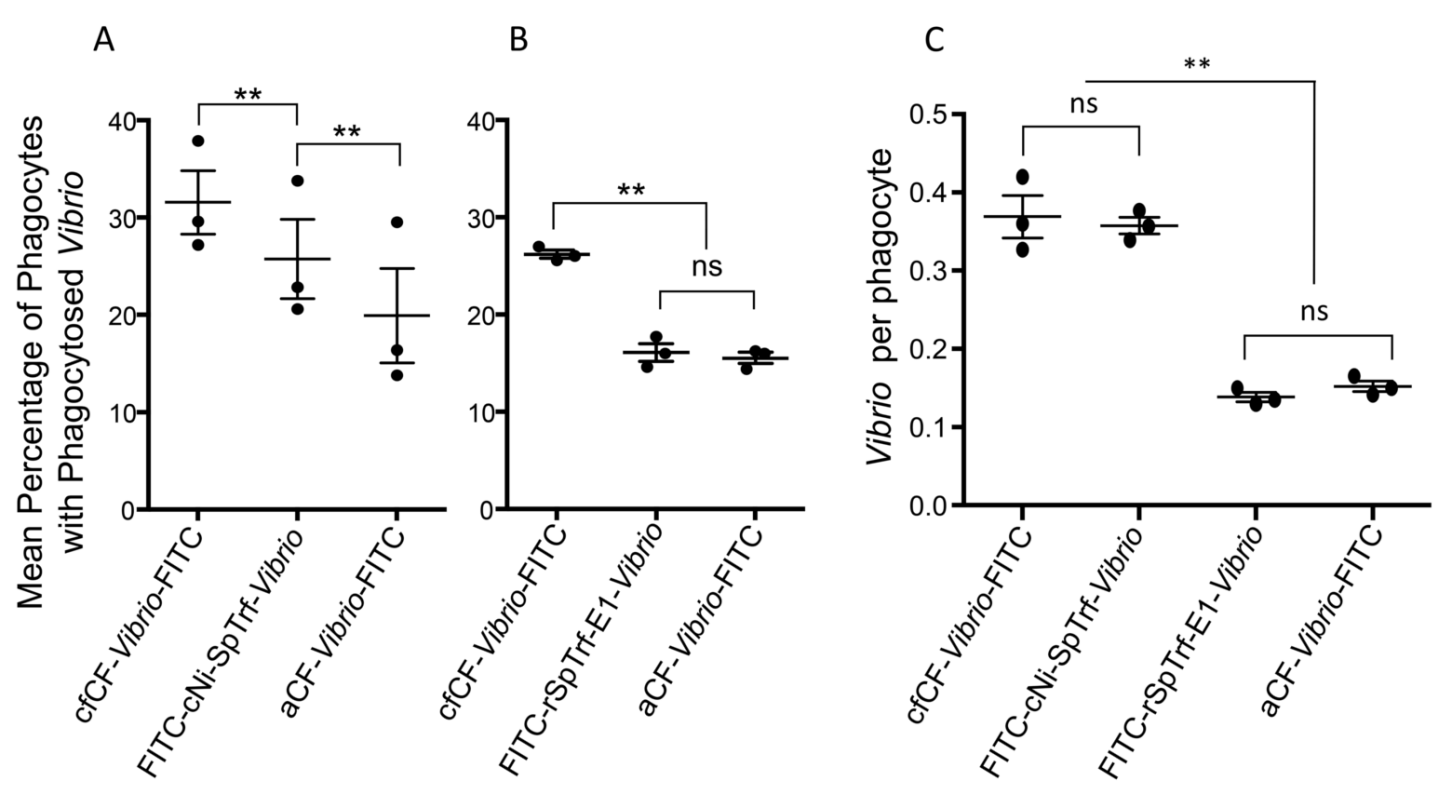
cNi-SpTrf proteins augment phagocytosis whereas rSpTrf-E1 proteins do not. **A**. Opsonized *Vibrio diazotrophicus* was incubated with phagocytes for 70 min followed by fixing, staining, and analysis of the mean percentage of phagocytes (n = 500) that contained *V*. *diazotrophicus*. cNi-SpTrf proteins from three different sea urchins were evaluated for opsonization. Phagocytes were obtained from a sea urchin that was different from those that provided the cNi-SpTrf proteins or the cfCF used for opsonization. The percentage of phagocytes with FITC-cNi-SpTrf-*Vibrio* is intermediate between number of phagocytes with cfCF-*Vibrio*-FITC (positive control) and aCF-*Vibrio*-FITC (buffer control) (see Table 1 for definitions of the abbreviations). **B**. Opsonization of *V*. *diazotrophicus* with SpTrf proteins (cfCF-*Vibrio*-FITC) isolated from three sea urchins results in a greater percentage of phagocytes with bacteria than either FITC-SpTrf-E1-*Vibrio* or aCF-*Vibrio*-FITC. **C**. *Vibrio diazotrophicus* opsonized with cNi-SpTrf, rSpTrf-E1, cfCF or aCF were incubated with coelomocytes at a ratio of 100 bacteria per phagocyte. Phagocytosis of FITC-cNi-SpTrf-*Vibrio* results in similar numbers of *V*. *diazotrophicus* per phagocyte compared to cfCF-*Vibrio*-FITC, and both show significantly greater numbers of bacteria per phagocyte than aCF-*Vibrio*-FITC. Phagocytosis of FITC-SpTrf-E1-*Vibrio* results in similar numbers of bacteria per phagocyte as that for aCF-*Vibrio*-FITC. **, statistically significant; ns, not significant.

The phagocyte class of coelomocytes is composed of large phagocytes (polygonal and discoidal) and small phagocytes (reviewed in [44]), and phagocytosis of yeast opsonized by the complement homologue, SpC3, is mediated by the large phagocytes in the purple sea urchin [63]. The initiation of phagocytosis of foreign cells requires a linkage, such an opsonin, between the microbe and the surface of the phagocyte to enable phagocytosis and incorporation into a phagosome [68, 69]. SpTrf proteins bind to bacteria and rSpTrf-E1 binds tightly to *V*. *diazotrophicus* [49] suggesting that these proteins may function as opsonins to augment phagocytosis. To test this, *V*. *diazotrophicus* was opsonized with cNi-SpTrf proteins isolated from three sea urchins followed by labeling with FITC (FITC-cNi-SpTrf-*Vibrio*; see Table 1) and incubation with phagocytes. Results showed that significantly more FITC-cNi-SpTrf-*Vibrio* were phagocytosed compared to aCF-*Vibrio*-FITC, the non-opsonized control (Fig 4A). However, the percentage of phagocytes with FITC-cNi-SpTrf-*Vibrio* was significantly less than those with FITC-stained *V*. *diazotrophicus* opsonized with cfCF (cfCF-*Vibrio*-FITC), which served as the positive control. The difference may have been due to cfCF that likely included a wide variety of opsonins and agglutinins compared to cNi-SpTrf proteins. Alternatively, when phagocytes were incubated with *V*. *diazotrophicus* opsonized with rSpTrf-E1 (FITC-rSpTrf-E1-*Vibrio*), the percentage of cells with bacteria was significantly less than cfCF-*Vibrio*-FITC and was not different from aCF-*Vibrio*-FITC (Fig 4B). When the average number of *V*. *diazotrophicus* cells taken up per phagocyte was evaluated, cNi-SpTrf-*Vibrio*-FITC and cfCF-*Vibrio*-FITC were present in similar numbers per phagocyte, which were significantly more than for aCF-*Vibrio*-FITC (Fig 4C). However, the numbers of FITC-rSpTrf-E1-*Vibrio* per phagocyte was not different from aCF-*Vibrio*-FITC. These results indicated that *V*. *diazotrophicus* opsonized with arrays of cNi-SpTrf proteins isolated from three sea urchins augmented phagocytosis but were not as effective as opsonins in the cfCF. Although rSpTrf-E1 is known to bind *V*. *diazotrophicus* [49], it did not demonstrate effective opsonization that led to augmented phagocytosis.

**Fig 5:**
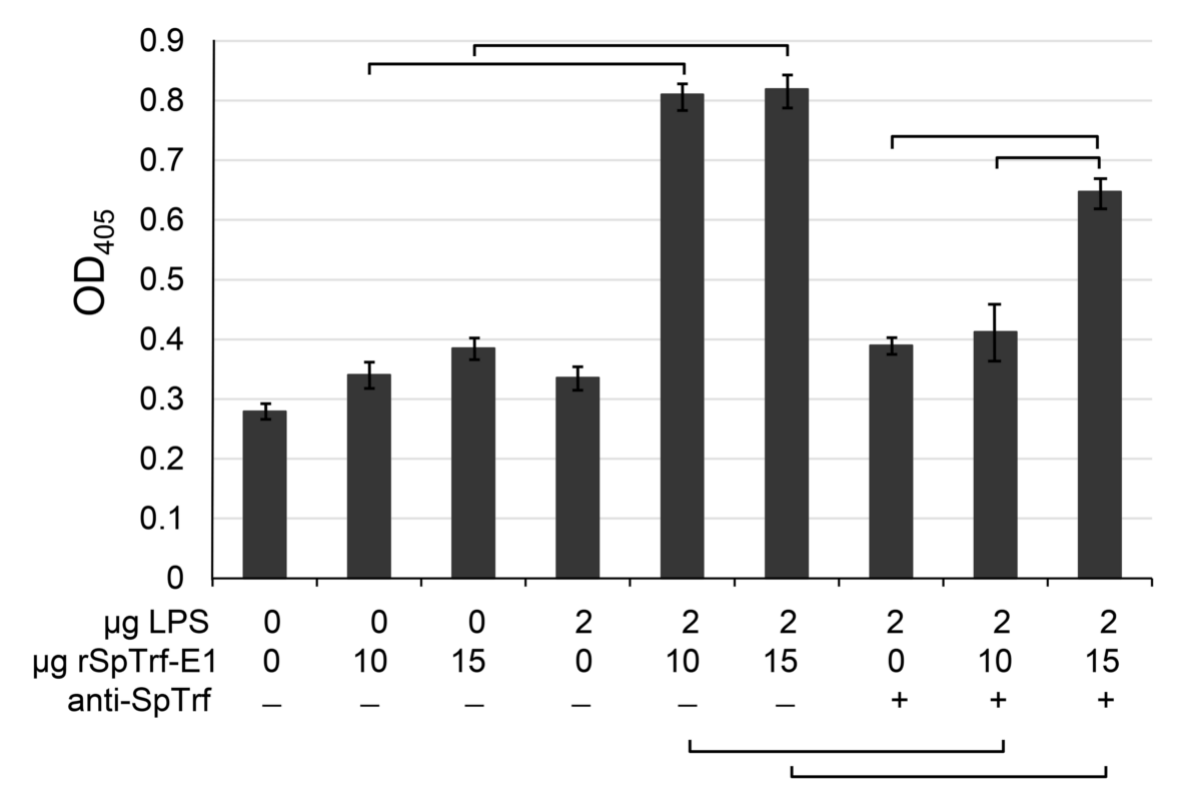
rSpTrf-E1 has binding specificity to LPS. Increasing concentrations of rSpTrf-E1 pre-bound to anti-SpTrf were analyzed by ELISA. In the absence of the antisera, significantly more rSpTrf-E1 is bound to LPS compared to the background controls in the absence of LPS. Pre-incubation of rSpTrf-E1 with anti-SpTrf antibodies decreases binding to LPS significantly compared to samples in which the antisera are omitted. Data are shown as the mean and ± standard deviation (SD) of three replicates. Brackets indicate significance (*p* < 0.05) using Student’s two-tailed paired *t* test with equal variance.

**Fig 6:**
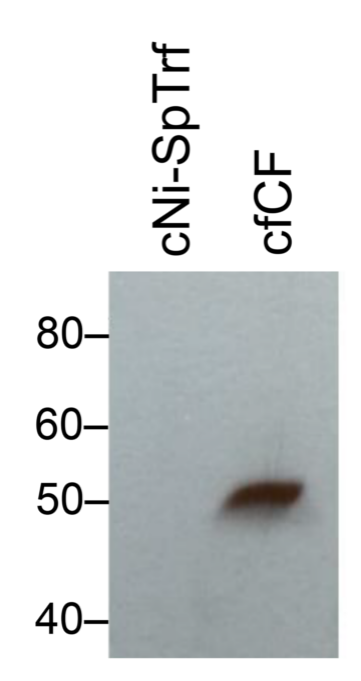
cNi-SpTrf proteins are not contaminated with detectible amounts of SpC3. Pelleted coelomocytes were sonicated, the lysate was passed through a nickel column, and an eluted fraction containing cNi-SpTrf proteins was evaluated by Western blot with anti-SpC3-6His [64]. A sample of cfCF that was not subject to nickel affinity was evaluated on the same filter for comparison. The cNi-SpTrf proteins do not contain detectable traces of SpC3 while the cfCF fraction has an autolytic fragment of the SpC3 α chain [70] indicating the presence of an active complement protein homologue.

**Fig 7:**
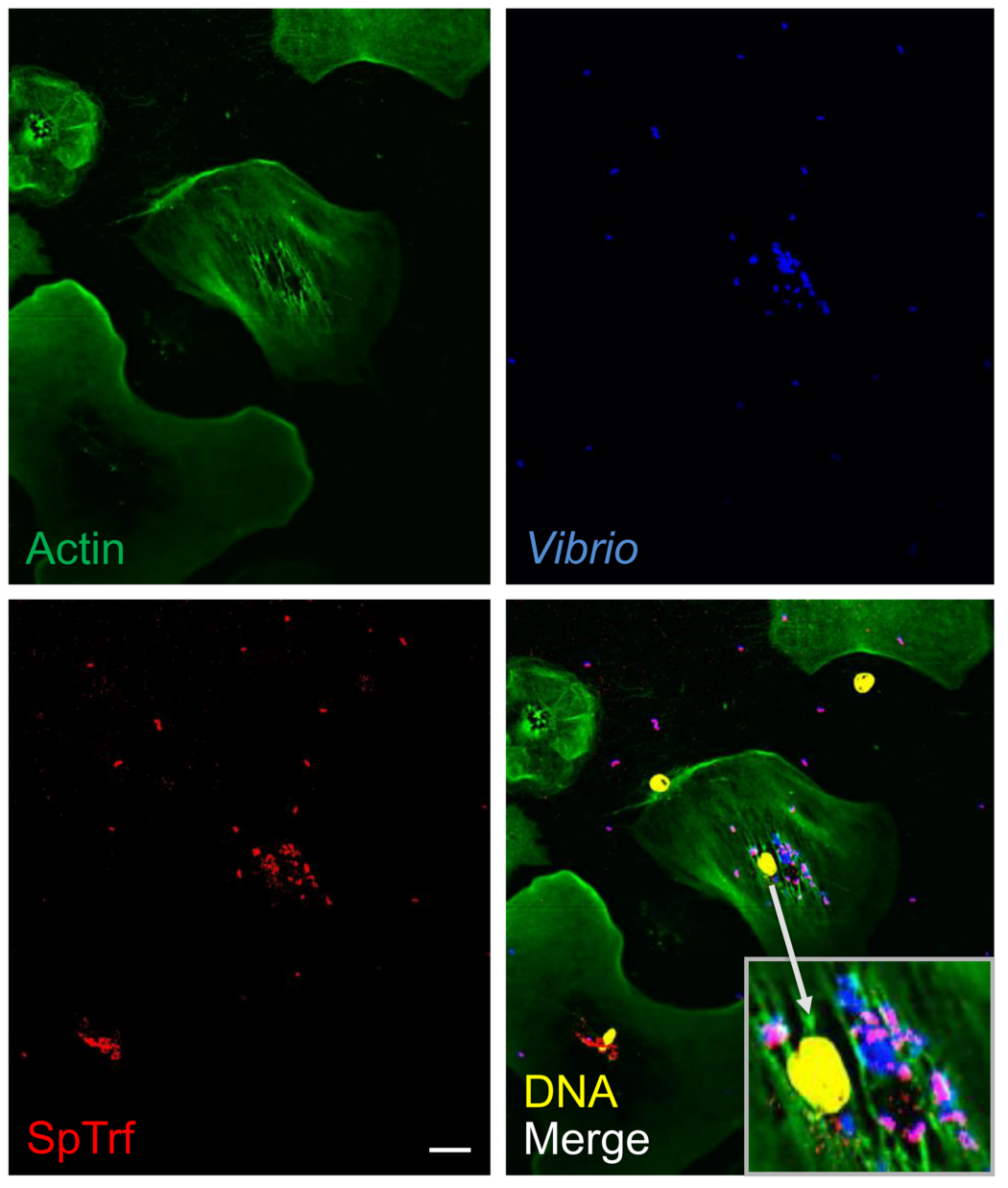
SpTrf proteins are secreted from coelomocytes in culture and opsonize *Vibrio diazotrophicus*. Non-opsonized, heat-killed *V*. *diazotrophicus* were incubated with immune-activated coelomocytes for 24 hr at 14°C followed by fixing and staining for fluorescence microscopy. Some of the phagocytosed *V*. *diazotrophicus* and almost all of the extracellular *V*. *diazotrophicus* are coated with SpTrf proteins. The insert in the lower corner of the merge panel is a magnification of the perinuclear region of the central polygonal phagocyte. The nuclear color of the phagocytes was altered using the confocal microscope imaging program. Some phagocyte nuclei become dissociated from the cells during processing. Scale bar indicates 10 μm.

To verify that rSpTrf-E1 was bound to *V*. *diazotrophicus* prior to the phagocytosis assay and to demonstrate binding specificity, rSpTrf-E1 binding to LPS and evaluated by ELISA was used as a proxy for binding specificity to Gram negative bacteria. Results indicated that rSpTrf-E1 bound to LPS at significantly higher levels compared to background in the absence of LPS (Figs 5 and 6). Furthermore, when anti-SpTrf antibodies were mixed with rSpTrf-E1 prior to incubation with LPS, binding was significantly decreased indicating binding specificity. The question of whether other opsonins were isolated by nickel affinity from the CF in parallel with the SpTrf proteins has been addressed previously, and the sea urchin complement homologue, SpC3, has been identified by tandem mass spectrometry (MS) [53]. To determine whether SpC3 was isolated by nickel affinity in this study, cNi-SpTrf proteins from immune challenged sea urchins were evaluated by Western blot and showed no detectable SpC3 (Figs 6 and 7). However, prior to nickel affinity isolation, a ~50 kDa autolytic fragment of the SpC3 α chain was identified in cfCF indicating the presence of SpC3 proteins in the CF with an active thioester motif [70]. The absence of SpC3 detection by Western blot was in agreement with a similar analysis of wNi-SpTrf proteins [53]. Because MS is highly sensitive to very small amounts of proteins, these conflicting results suggested that the concentration of SpC3 and other immune related proteins, including several versions of scavenger receptor cysteine-rich proteins and α-2 macroglobulin, were likely either below the level required for functional activity in the Ni-isolated fraction or did not have opsonization activity. Finally, when SpTrf proteins were isolated first by nickel affinity followed by anti-SpTrf antibody affinity, opsonization and phagocytosis for these double-isolated proteins were not different from cNi-SpTrf proteins (data not shown). Overall, these data suggested that cNi-SpTrf proteins functioned as opsonins and augmented phagocytosis of bacteria.

### SpTrf proteins are secreted from coelomocytes

**Fig 8:**
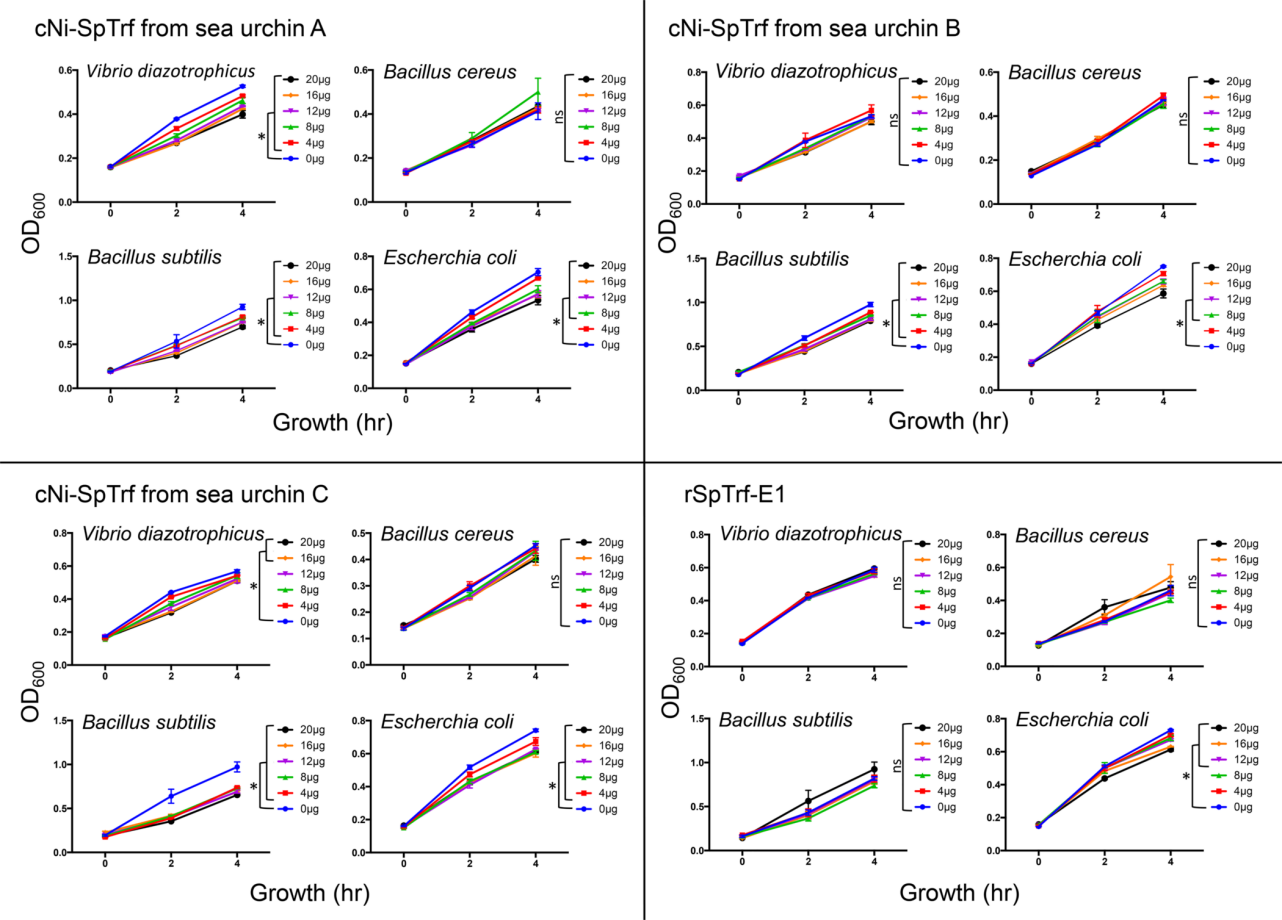
SpTrf proteins and rSpTrf-E1 show partial activity to retard bacterial growth. *Vibrio diazotrophicus*, *Bacillus cereus*, *B*. *subtilis*, or *Escherichia coli* were grown to OD_600_ = 1, and 10 μl were incubated with increasing concentrations of cNi-SpTrf proteins isolated from sea urchins A, B, and C, or with increasing concentrations of rSpTrf-E1. Bacterial growth was determined in triplicate by measuring the turbidity by OD_600_ at 0, 2, and 4 hr. Data are presented as growth or OD_600_ relative to time in the presence of increasing amounts of SpTrf proteins (μg/200 μl). The mean background OD_600_ for the blank wells without bacteria was subtracted from the corresponding experimental time points. Student’s one-tailed *t* test was used to evaluate the significance of change in turbidity in the absence of SpTrf proteins compared to the range of SpTrf protein concentrations added to the cultures. Statistical significance is indicated by **, *p* ≤ 0.005; *, *p* ≤ 0.05. ns, not significant.

SpTrf proteins are thought to be strictly associated with cells because they have only been observed in association with membranes of perinuclear vesicles of phagocytes and with the plasma membrane and cell surface of small phagocytes [46, 52, 60]. We have assumed that the SpTrf proteins are not secreted because they are primarily associated with coelomocytes pelleted from wCF and are generally present in the cfCF in very low or undetectable levels (Smith, unpublished observation). However, if SpTrf proteins are strictly associated with membranes, this does not fit with bacterial binding and opsonization of extracellular microbes by cNi-SpTrf reported above. To address this question, non-opsonized, heat-killed *V*. *diazotrophicus* were incubated with glass-adherent coelomocytes collected from a sea urchin 24 hr after it had been immune activated by injections with heat-killed *V*. *diazotrophicus*. When the coelomocytes were incubated for 24 hr in culture with heat-killed *V*. *diazotrophicus*, most of the non-phagocytosed bacteria and some of the phagocytosed bacteria were coated with SpTrf proteins (Figs 7 and 8). This demonstrated that SpTrf proteins were produced by the phagocytes in culture, which were secreted into the medium and bound to the extracellular *V*. *diazotrophicus*. The internalized bacteria that were coated with SpTrf proteins may have been opsonized by the secreted SpTrf proteins followed by phagocytosis. Alternatively, some bacteria may have been phagocytosed first, followed by the fusion of perinuclear vesicles containing SpTrf proteins with the phagolysosomes and subsequent binding of the SpTrf proteins to the bacteria. Previous work has suggested that extreme changes in pH does not alter the binding activity of the SpTrf proteins to bacterial target cells [71]. Because SpTrf proteins are generally not observed in the cfCF, these results suggested that *in vivo*, the proteins may be secreted upon detection of foreign cells to which they may bind quickly and are essentially removed from the cfCF. Hence they may only be present transiently in the cfCF.

### cNi-SpTrf proteins retard bacterial growth whereas rSpTrf-E1 does not

**Fig 9:**
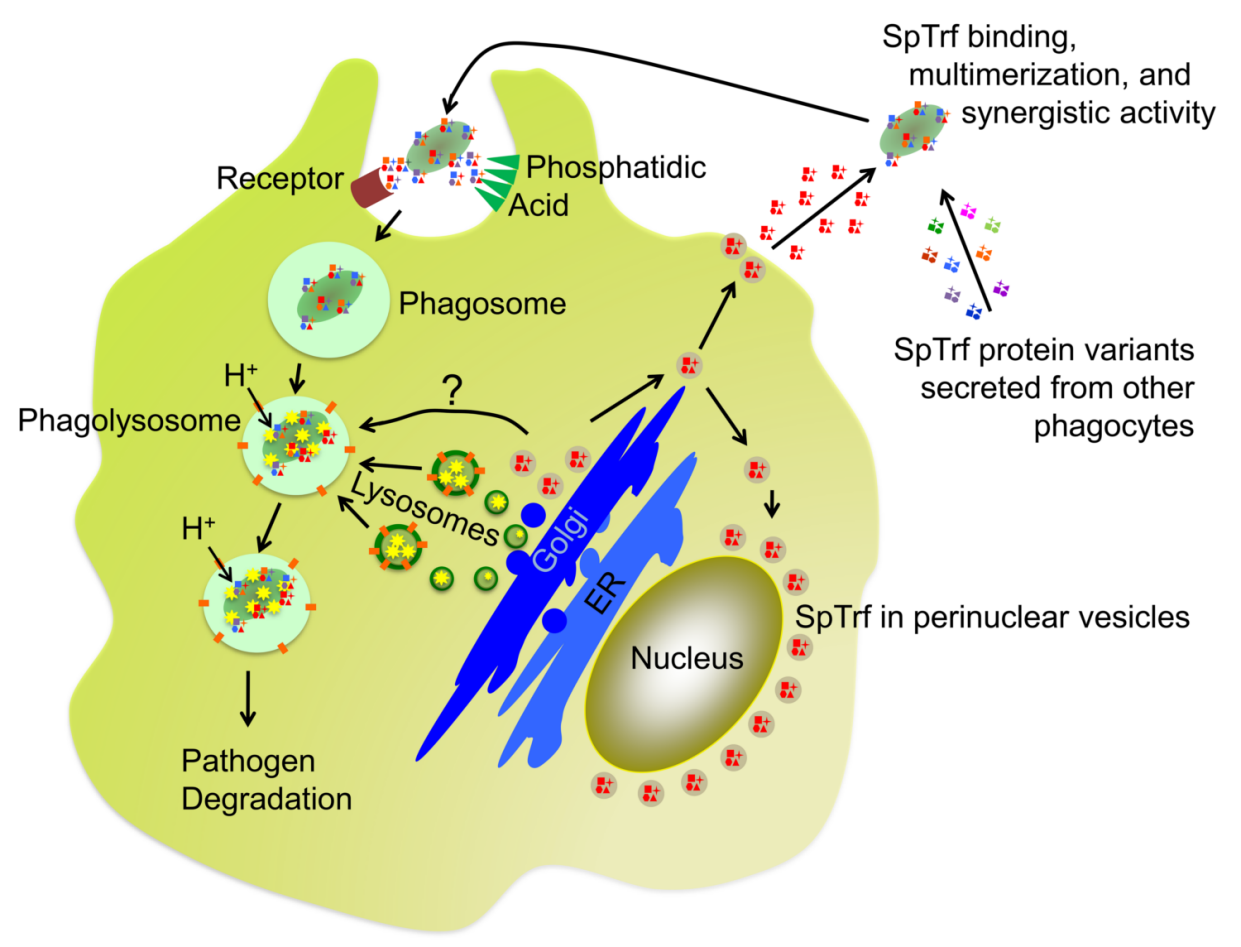
Putative trafficking and function of SpTrf proteins. The current hypotheses of SpTrf protein function is that they are initially produced and stored in perinuclear vesicles of phagocytes. Note that a single *SpTrf* gene is expressed in a single phagocyte according to Majeske et al. [47], which would produce a few versions of the SpTrf protein (shown in red) resulting from RNA editing [54] and post translational processing [53]. SpTrf proteins are secreted from vesicles upon bacterial detection but vesicles are also hypothesized to fuse with phagolysosomes and SpTrf proteins may function in the degradation of phagocytosed microbes. After secretion, multiple SpTrf isoforms (indicated in multiple colors) mix in the CF and multimerize upon opsonization of bacteria that augments phagocytosis and may retard bacterial growth. How phagocytes recognize SpTrf proteins on the surface of microbes is not known, however, a speculative receptor (brown) is shown. SpTrf proteins may also bind to phosphatidic acid (PA; green triangles), which has been demonstrated for SpTrf-E1 [56]. The conical structure of PA and its clustering induced by SpTrf binding and multimerization is known to drive membrane curvature, which may augment phagocytosis. ER, endoplasmic reticulum.

cNi-SpTrf proteins bind to *V*. *diazotrophicus*, *Bacillus cereus*, *B*. *subtilis*, and *E*. *coli*, and rSpTrf-E1 shows strong, specific, irreversible binding to LPS (Figs 5 and 6) and *V*. diazotrophicus [49]. Hence, these results suggested that cNi-SpTrf proteins and rSpTrf-E1 may have direct activity on bacteria in the absence of coelomocytes. cNi-SpTrf proteins isolated from three sea urchins were incubated with bacteria and growth was evaluated by monitoring culture turbidity at 0, 2 and 4 hr. Results showed that for some combinations, retarded bacterial growth correlated with increasing concentrations of cNi-SpTrf proteins (Figs 8 and 9). Proteins from all three sea urchins retarded the growth of *B*. *subtilis*, and *E*. *coli*, whereas only proteins from animals A and C retarded the growth of *V*. *diazotrophicus*. None of the cNi-SpTrf proteins isolated from any of the sea urchins retarded the growth of *B*. *cereus*. These results demonstrated that cNi-SpTrf proteins had partial activity to slow bacterial growth, and that the proteins isolated from different animals had different levels of activity on different species of bacteria. Higher concentrations of rSpTrf-E1 retarded the growth of *E*. *coli* but had no significant effect on the other microbial species (Figs 8 and 9). These results were consistent with the report that rSpTrf-E1 does not bind to *Bacillus* spp [49] and indicated that although rSpTrf-E1 binds tightly to *V*. *diazotrophicus*, it did not alter growth. Results from both phagocytosis and growth retardation activity suggested that different isoforms of the SpTrf proteins may have different functions.

## Discussion

cNi-SpTrf proteins isolated from pelleted coelomocytes show increased binding to target cells with increased concentrations of proteins leading to saturation plateaus consistent with a finite number of specific binding sites. These results are in agreement with the binding characteristics of rSpTrf-E1 to *V*. *diazotrophicus*, *S*. *cerevisiae*, and several PAMPs [49]. cNi-SpTrf proteins isolated from different sea urchins show different patterns of binding to microbes, in agreement with preliminary results for wNi-SpTrf proteins [53]. These results suggest that there are variations in the SpTrf isoforms expressed by individual sea urchins including possible variations in their expression levels, which may correlate with differences in the members of the *SpTrf* gene family among animals [38], and is an additional level of diversity for this system. cNi-SpTrf opsonization of *V*. *diazotrophicus* augments phagocytosis but is not as effective as opsonization by proteins in the cfCF of immune activated sea urchins. Although rSpTrf-E1 binds *V*. *diazotrophicus* (shown here and see [49]), it does not augment phagocytosis, which is a basis for suggesting that isoforms with different sequences have different functions. The cNi-SpTrf proteins have partial activity to retard growth, depending on the animal from which they are isolated and the species of bacteria with which they are tested. The rSpTrf-E1 proteins show more limited activity to retard growth for fewer bacterial species. SpTrf proteins are secreted from phagocytes, rather than remaining bound to membranes, and opsonize *V*. *diazotrophicus*. These results for the subset of SpTrf proteins that can be isolated by nickel affinity indicate a wide range of activities that varies among proteins and among individual sea urchins.

### Multiple levels of SpTrf diversification

There are multiple levels at which diversification of the *SpTrf* system are thought to occur (reviewed in [72]). i) *SpTrf* gene sequences may be diversified through genomic instability that is the outcome of tight clusters, segmental duplications, multiple types of repeats, shared sequences among genes, and short tandem repeats surrounding each gene. The diversity is so great among the genes that identical gene sequences have not been identified among animals [48]. ii) Variations in gene expression may underlie, in part, changes in the protein arrays. iii) Message sequence diversity appears to be augmented through RNA editing [54]. iv) The outcome of message editing results in the translation of full-length proteins with increased amino acid diversity, and proteins with missense and/or truncated sequence that commonly deletes the histidine-rich region [35, 52]. v) Protein diversity may be increased by putative post-translational modifications [53]. Diverse arrays of SpTrf proteins are expressed by phagocytes that show a broad range of pI and molecular weight and change in response to immune challenge [52, 53], which results in dynamic expression that varies among individual sea urchins. Changes in protein arrays over time is in agreement with the changes in the sizes of the cDNA amplicons that have been observed in response to PAMP challenge over time [35]. Multitasking activities for individual proteins is based on results for rSpTrf-E1 evaluated in the absence of other sea urchin proteins, which binds to *V*. *diazotrophicus*, *S*. *cerevisiae*, LPS, non-glycosylated flagellin from *Salmonella*, the phospholipid, phosphatidic acid (PA), and β-1,3-glucan, all likely through conformational changes from disordered to α helical [49, 55, 56]. Hence, the *SpTrf* system appears to be an extreme example of multiple levels of diversification that is likely driven by the arms race between sea urchins and the multitudes of marine microbes. Optimization of the system is an outcome of co-evolution through selection for effective host defense mechanisms in competition with diversification mechanisms for improved virulence, proliferation, and survival of the pathogens [1].

### Synergistic function of SpTrf proteins

rSpTrf-E1 binds tightly to *V*. *diazotrophicus*, however it does not induce phagocytosis nor does it display growth retardation activity towards this bacterial species. Although rSpTrf-E1 does not bind to *Bacillus* spp. or to PGN [49], mixtures of cNi-SpTrf proteins bind to both *V*. *diazotrophicus* and the two *Bacillus* species. The comparison of the activities between a single SpTrf protein functioning alone and multiple SpTrf isoforms functioning together supports the notion that different SpTrf isoforms have different functions and that they may function synergistically when secreted into the cfCF from phagocytes. This idea suggests a sixth level of diversification for the SpTrf system through variations in protein-protein interactions and multimerization. The frequency of cDNA sequences encoding a particular element pattern before vs. after immune challenge is variable among animals and with regard to the type of immune challenge [35], and may correlate with the functions of the various SpTrf isoforms. We speculate that rare SpTrf variants such as SpTrf-E1 [36] may have narrower binding specificities for foreign cells with limited anti-bacterial activities. To induce phagocytosis, SpTrf-E1 would require synergistic activities of other isoforms for functional opsonization of *V*. *diazotrophicus*. There are likely other activities of the SpTrf proteins besides growth retardation activity shown here, and may include bactericidal functions that have been hypothesized from the highly lethal expression of most recombinant SpTrf proteins in *E*. *coli* [49]. The results reported here must be considered in light of the fact that isoforms with insufficient histidines or those that are truncated and only include the glycine-rich region and the multimerization region (Fig 1) are not isolated with this approach and therefore not included in this analysis. However, analysis of cDNA prevalence before vs. after immune challenge indicates an increase in full length proteins with the histidine-rich region [35, 53]. In general, the various isoforms of SpTrf proteins may have multitasking and overlapping activities for binding bacteria and PAMPs including variable capabilities for inducing phagocytosis. Our results lead us to speculate that depending on the pathogen that infects sea urchins, these invertebrates are capable of expressing and secreting arrays of SpTrf variants that function synergistically and efficiently to target pathogens for removal and destruction by phagocytes for optimal host protection.

### *SpTrf* gene expression in single phagocytes

The diversity of the SpTrf system suggested by functional variations among different SpTrf protein isoforms and the multimerization among proteins to integrate and optimize their functional variations, must include the unexpected *SpTrf* gene expression patterns from the phagocyte class of coelomocytes. The *SpTrf* transcripts from single phagocytes have identical sequence suggesting expression from a single member of the *SpTrf* gene family per cell [47]. This infers that each cell may secrete a limited number of very similar SpTrf proteins with the same element pattern but that are somewhat variable based on mRNA editing [54] and putative post-translational modifications [52, 53]. Differences in the arrays of SpTrf proteins among individual sea urchins responding to different microbial challenges [53], plus the notion that some SpTrf protein isoforms may have broad spectrum activities and that other isoforms may have much more limited functions, suggests a complex pathogen detection system and a complex system for regulating *SpTrf* gene expression in sea urchin phagocytes [47]. These changes may correlate with changes in the percentages of small and polygonal phagocytes that express SpTrf proteins in response to LPS [46]. We show that multiple phagocytes in short term cultures secrete SpTrf proteins that bind to *V*. *diazotrophicus* located both outside of the cells and within phagosomes. However, the advantages of expressing a single *SpTrf* gene per single cell and secreting a highly similar isoforms is perplexing as this expression pattern does not appear to be optimal for swift production and secretion of these proteins in large quantities in response to pathogen invasion. We speculate that packing homogeneous SpTrf proteins into transport vesicles may in some way aid in blocking multimerization that maintains activity. We have shown that once multimerized, SpTrf proteins do not return to monomers, that multimerized SpTrf proteins do not bind to microbes (Chou, Lun, Smith, unpublished observations), and that multimerized SpTrf and rSpTrf-E1 do not bind to PA [56]. Multiple isoforms present in the CF likely lead to the formation of multimeric complexes after binding to foreign cells, which may promote phagocytosis and perhaps other anti-microbial activities. Synergistic multimers would be expected to have advantages for sea urchins because the mix-and-match multimerization may broaden the functional capabilities of individual SpTrf proteins for binding to a variety of microbes and adds an additional layer of complexity to this system through SpTrf protein-protein interactions.

## Conclusion

Here we report the diversity and functions of SpTrf proteins that are produced and secreted by phagocytes and have sufficient histidines for isolation by nickel affinity. We compared their activities to a single recombinant SpTrf protein, rSpTrf-E1. The SpTrf system demonstrates the complexity and sophisticated nature of the innate immune system of the purple sea urchin, which acts at multiple levels, including multiple targets for some or all isoforms and putative synergism among proteins to form complexes that may broaden the activities of the individual proteins. SpTrf proteins are present in vesicles near the nuclei of phagocytes, which are secreted from single phagocytes as proteins with a single element pattern (Fig 9). Many isoforms secreted from many phagocytes in the CF are predicted to bind to microbes that augment phagocytosis and may retard microbial growth independent of phagocyte function. We propose that the SpTrf proteins multimerize on the surface of the microbe, based on results for rSpTrf-E1 that binds to and clusters PA in liposomes, which is a likely outcome of protein multimerization [56]. The phagocyte cell surface receptor for SpTrf proteins bound to bacteria is not known, but may include a variety of macromolecules with exposed phosphate groups, which would be bound by the histidine-rich region of the proteins. The conical shape of PA [73] induces membrane curvature when clustered, a characteristic that may correlate with membrane curvature during phagocytosis (Fig 9). In addition to transport to the plasma membrane for secretion, perinuclear vesicles carrying SpTrf proteins may also deliver their cargo to the endosomal system for fusion with phagolysosomes. We speculate that the SpTrf proteins may participate downstream of phagocytosis to block pathogen proliferation and perhaps aid in pathogen degradation. Overall, these mechanisms may protect sea urchins from a very broad spectrum of pathogens that are bound and removed efficiently from the CF. The SpTrf system demonstrates diversity, flexibility, and efficiency of antimicrobial activity, which is an important aspect for survival in the marine environment and the longevity of purple sea urchins.

## Supporting information

S1 Fig rSpTrf-E1 binds to *Vibrio diazotrophicus* and *Saccharomyces cerevisiae* with saturable kinetics. (**A**) *Vibrio diazotrophicus* (2.9 X 10^8^ cells) or (**B**) *Saccharomyces cerevisiae* (1.48 X 10^4^ cells) were incubated in three trials each with increasing concentrations biotinylated rSpTrf-E1 for 30 min at 14°C. Control bacteria were incubated in standard PBS without biotinylated rSpTrf-E1. After incubation, bacteria were washed three times in PBS and incubated in 0.1% NeutrAvidin fluorescein isothiocyanate (NeuFITC) conjugate (Invitrogen) in 500 μl of PBS for 30 min at 14°C. Bacteria were washed three times, resuspended in 200 μl of PBS and loaded in triplicate into wells of a black, round-bottom 96-well assay plate (Corning Life Sciences). Fluorescence was detected with a Synergy HT Multi-Mode Microplate Reader (excitation/emission: 490/525) and analyzed with Gen5 Data Analysis Software (BioTek). The background level for each experiment was based on the fluorescence level of NeuFITC that bound directly to *V*. *diazotrophicus* or *S*. *cerevisiae* in the absence of biotinylated rSpTrf-E1. For both types of target cells, binding of FITC-rSpTrf-E1 (see Table 1 in the main paper for definitions of abbreviations) shows a saturation plateau in agreement with Lun et al. [49] that was observed by flow cytometry for this type of analysis. Means and standard deviation are shown.
(TIF)
