# Supplementary figures and images for: SpTransformer proteins from the purple sea urchin opsonize bacteria, augment phagocytosis, and retard bacterial growth
Source: PLoS One. 2018 May 8;13(5):e0196890. doi: 10.1371/journal.pone.0196890 (PMC5940198; doi:10.1371/journal.pone.0196890)

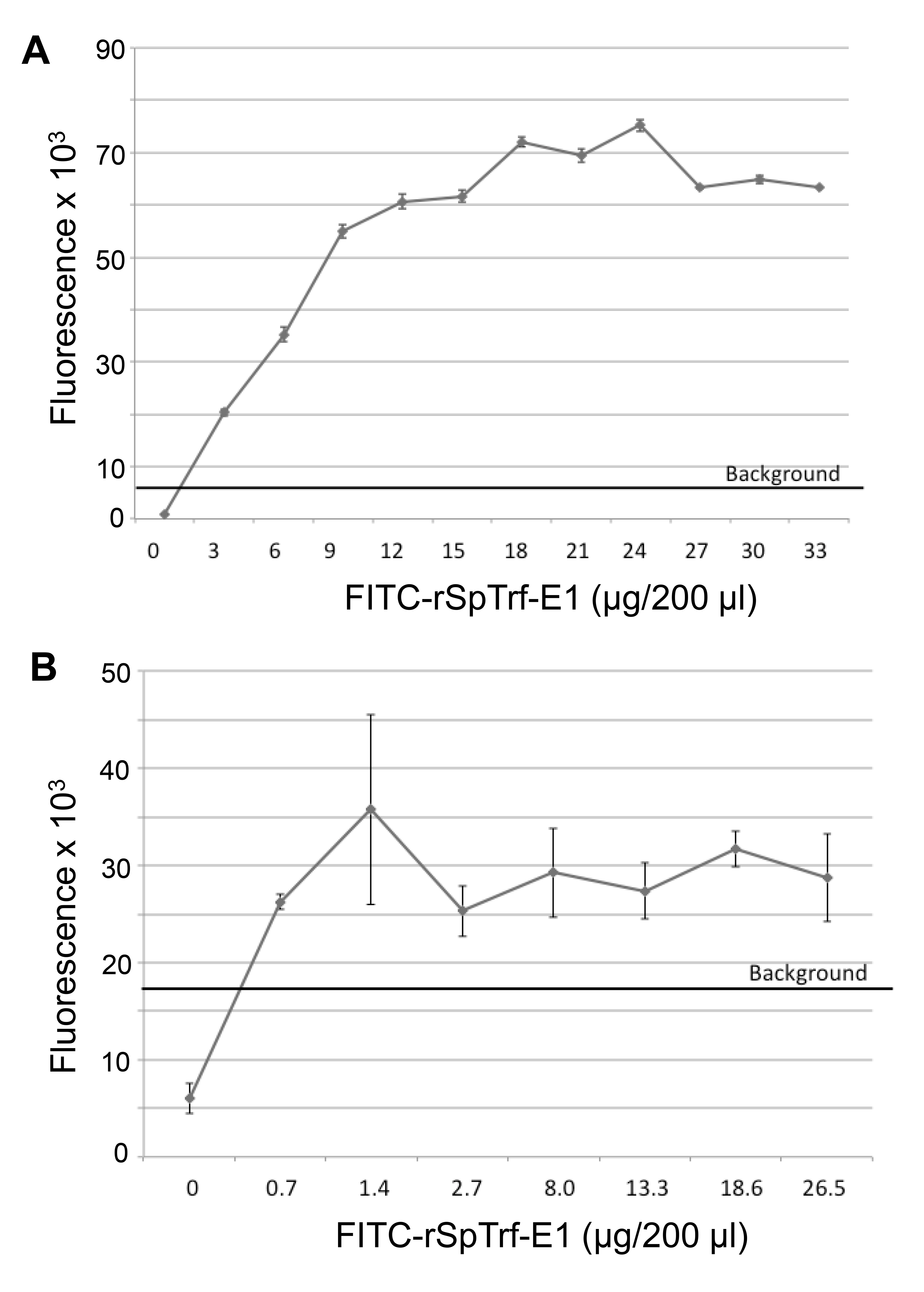

Supplement: S1 Fig — (A) Vibrio diazotrophicus (2.9 X 108 cells) or (B) Saccharomyces cerevisiae (1.48 X 104 cells) were incubated in three trials each with increasing concentrations biotinylated rSpTrf-E1 for 30 min at 14°C. Control bacteria were incubated in standard PBS without biotinylated rSpTrf-E1. After incubation, bacteria were washed three times in PBS and incubated in 0.1% NeutrAvidin fluorescein isothiocyanate (NeuFITC) conjugate (Invitrogen) in 500 μl of PBS for 30 min at 14°C. Bacteria were washed three times, resuspended in 200 μl of PBS and loaded in triplicate into wells of a black, round-bottom 96-well assay plate (Corning Life Sciences). Fluorescence was detected with a Synergy HT Multi-Mode Microplate Reader (excitation/emission: 490/525) and analyzed with Gen5 Data Analysis Software (BioTek). The background level for each experiment was based on the fluorescence level of NeuFITC that bound directly to V. diazotrophicus or S. cerevisiae in the absence of biotinylated rSpTrf-E1. For both types of target cells, binding of FITC-rSpTrf-E1 (see Table 1 in the main paper for definitions of abbreviations) shows a saturation plateau in agreement with Lun et al. [49] that was observed by flow cytometry for this type of analysis. Means and standard deviation are shown. (TIF) [file pone.0196890.s001.tif]
